# Rethinking the bile acid/gut microbiome axis in cancer

**DOI:** 10.18632/oncotarget.22803

**Published:** 2017-12-01

**Authors:** John P. Phelan, F. Jerry Reen, Jose A. Caparros-Martin, Rosemary O'Connor, Fergal O'Gara

**Affiliations:** ^1^ BIOMERIT Research Centre, School of Microbiology, University College Cork - National University of Ireland, Cork, T12 YN60, Ireland; ^2^ School of Biochemistry and Cell Biology, University College Cork, National University of Ireland, Cork, T12 YN60, Ireland; ^3^ Human Microbiome Programme, School of Biomedical Science, Curtin Health Innovation Research Institute, Curtin University, Perth, WA 6102, Australia

**Keywords:** bile acids, microbiome, gut-axis, cancer, dysbiosis

## Abstract

Dietary factors, probiotic agents, aging and antibiotics/medicines impact on gut microbiome composition leading to disturbances in localised microbial populations. The impact can be profound and underlies a plethora of human disorders, including the focus of this review; cancer. Compromised microbiome populations can alter bile acid signalling and produce distinct pathophysiological bile acid profiles. These in turn have been associated with cancer development and progression. Exposure to high levels of bile acids, combined with localised molecular/genome instability leads to the acquisition of bile mediated neoplastic alterations, generating apoptotic resistant proliferation phenotypes. However, in recent years, several studies have emerged advocating the therapeutic benefits of bile acid signalling in suppressing molecular and phenotypic hallmarks of cancer progression. These studies suggest that in some instances, bile acids may reduce cancer phenotypic effects, thereby limiting metastatic potential. In this review, we contextualise the current state of the art to propose that the bile acid/gut microbiome axis can influence cancer progression to the extent that classical *in vitro* cancer hallmarks of malignancy (cell invasion, cell migration, clonogenicity, and cell adhesion) are significantly reduced. We readily acknowledge the existence of a bile acid/gut microbiome axis in cancer initiation, however, in light of recent advances, we focus exclusively on the role of bile acids as potentially beneficial molecules in suppressing cancer progression. Finally, we theorise that suppressing aggressive malignant phenotypes through bile acid/gut microbiome axis modulation could uncover new and innovative disease management strategies for managing cancers in vulnerable cohorts.

## INTRODUCTION

In the 1940s, bile acids were shown to be inducers of cancer in rodents subcutaneously injected with the secondary bile acid, deoxycholate (DCA) [1]. This generated malignant spindle-celled tumours, indicative of epitheliomas (benign growths or malignant carcinomas that are classified according to the epithelial cell of origin). These early observations were correlatively affirmed in epidemiological studies that showed associations between bile acids and cancer, notably colorectal cancer (summarised in [2]). Since then, extensive research has revealed that bile acids act as tumour promoters in a variety of organs such as the oesophagus, the liver and the stomach lining [3, 4].

However, in recent years, several reports have emerged demonstrating the effectiveness of bile acids in reducing cell proliferation and migration of some cancer cell types. These observations have expanded our knowledge base in the understanding of bile acid signalling in the aetiology and evolution of cancer. However, we must also consider new and significant developments that have shaped our understanding of bile acid metabolism, namely the microbiome. Through the exploration of contemporary developments in this area, we can begin to redefine the bile acid/microbiome axis and its putative role in tumour suppression.

### Bile acids as emerging molecules in tumour suppression

Bile acids are key signalling molecules that play important roles as emulsifiers in digestion and the absorption of dietary lipids [2, 5–7]. The evidence implicating bile acids in cancer (oesophageal, stomach, gut) has been extensively documented throughout the literature [3, 8, 9]. A combination of mechanisms appears to be responsible for bile induced cancer in organs where elevated bile acid levels persist, e.g. oxidative damage (ROS), epithelium proliferation, cell death, signalling activation and localised DNA instability [6]. Specifically, at the colon, bile acid induced mitochondrial dysfunction leads to oxidative damage, ROS accumulation and localised cell membrane damage [10, 11]. Similarly, DCA has been shown to induce epithelium hyper-proliferation via the activation of epidermal growth factor receptor (EGFR), extracellular signal-regulated kinase (ERK) and protein kinase C (PKC) signalling pathways [11]. Prolonged exposure to bile acids at the colon can also increase apoptosis and apoptosis resistance, via mitochondrial stress and ROS release, activation of cytochrome C and members of the Bcl-2 family [11, 12]. These mechanisms, and others (e.g. mitosis disruption, G1/G2 cell cycle arrest, endoplasmic stress) lead to chromosomal instability and DNA damage, underpinning the initial stages of colorectal tumorigenesis, thereby aggravating an already unstable environment [2, 6, 11, 13, 14, 15, 16].

The macro-level damage described above is also associated with significant changes in several key signalling pathways (Table 1). Constitutive activation and translocation of the transcription factor NF-κB, a protein involved in DNA transcription, inflammatory cytokine production and enhanced cell survival, occurs in response to DCA exposure at the oesophagus and colon [6, 14, 17–19]. Similarly, expression of cyclooxygenase, COX2 and prostaglandin E2 (PGE2) (a member of the prostaglandin family involved in inflammation, cell growth and hormone regulation) is significantly elevated in pancreatic cancer cell lines (BxPC-3 and SU 86.86) in response to DCA and CDCA [20]. COX2, cited as a stress marker induced in Barret’s mucosa (a predictor of oesophageal adenocarcinoma), is increased upon exposure to DCA and chenodeoxycholate (CDCA) in squamous cancer oesophageal cells. Interestingly, in the same study, a key regulator of squamous regulation p63, was rapidly decreased upon exposure to DCA, suggesting p63 could be lost in the transition between squamous and metaplasia phenotypes when exposed to bile acids [21].

**Table 1:** The impact of bile acids on signalling pathways involved in cancer aetiology

Bile Acid	Signal Pathway/Molecular basis	Bile acids mediated cancer	Reference
**DCA**	ROS mediated NF-κB activation	Oesophageal Cancer	Jenkins *et al* (2008)
**DCA (plus aspirin)**	NF-κB activation	Gastric apoptosis (limited by aspirin)	Redlak MJ *et al* (2008)
**Bile Acids**	ROS, DNA damage, inflammation, NF-κB activation, enhanced cell proliferation	Colon cancer	Payne CM *et al* (2007), Jenkins *et al*
**CA**	Activates Cdx2 promoter via NF-κB	Metaplasia leading to Barrets' oesophagus	Kazumori *et al* (2006)
**CDCA and DCA**	Targets COX2 (stress responder) and p63 (transcriptional regulator)	Squamous to metaplasia cell transition in Oesophageal Cancer	Roman S *et al* (2007)
**Bile acids**	Deregulation of TSC1/mTOR by bile acids	Oesophageal adenocarcinoma	Yen *et al* (2008),
**DCA**	ROS induction, DNA damage (dose dependent) MUC2 induction	Oesophageal Adenocarcinoma and DCA	Jenkins GJ *et al* (2008) Wu J *et al* (2008)
**DCA**	DCA combined with methylselenol inhibits colon cancer cell proliferation. Induces SAPK/JNK1/2, p38 MAPK, ERK1/2 activation	Colon Cancer	Zeng *et al* (2010)
**DCA**	DCA targets cell proliferation and decreases suppressor activation in cell cycle and apoptosis. Activates ERK1/2, caspase-3 and PARP	Colon Cancer	Zeng *et al* (2015)
**DCA**	ROS induced genotoxicity in OE33 cells	Oesophageal Cancer	Jenkins *et al* (2007)

The major target of bile signalling appears to be NF-κB which is activated in several cancers including colon and oesophageal cancer.

Notwithstanding these observations, it has been shown that key *in vitro* cell morphological characteristics of cancer cells (e.g. cell invasion, cell migration, cell adhesion and cell survival) can be targeted by bile acids to suppress metastatic phenotypes in several cancer models (Table 2). In the leukaemia cell line HL60, DCA, CDCA and lithocholic acid (LCA) inhibit cell proliferation and induce cell differentiation with a mechanism implicating protein kinase C (PKC) [23]. Similarly, in HL60 cells and the leukaemia cell line THP-1, bile acids induce a positive cooperativity with retinoic acid and vitamin D in promoting differentiation, and down-regulating the serine protease myeloblastin. The expression of myeloblastin is associated with the promotion of cellular differentiation in myeloid leukaemia [22–24]. The proliferative capacity of the pancreatic cell lines, PANC-1, MIA PaCa-2 and PGHAM-1 have been tested in response to DCA. Cell proliferation was reduced and structural alterations to microvilli were observed in some cell lines, suggesting DCA could limit *in vitro* pancreatic cancer cell progression and invasion [25]. DCA and CDCA destabilised the transcription factor, Hypoxia Inducible Factor-1α (HIF-1α) in the immortalised bronchial epithelial cell line IB3-1 derived from a cystic fibrosis (CF) patient [26]. Similarly, DCA was shown to inhibit cell invasion, cell migration and cell proliferation in the gastric carcinoma cell lines SNU-216 and MKN45 [27]. This was accompanied by simultaneous reductions in Snail and MMP9 expression, proteins which are associated with aggressive menenchymal cell phenotypes. Similarly, increases in MUC2 and E-cadherin expression were also recorded and are associated with less aggressive cancers. Critically, these observations were used in a retrospective study to clinically evaluate tumour samples from a cohort with good cancer prognoses. MUC2 positive tumours showed reduced Snail expression, suggesting bile acids could regulate tumour behaviour in oesophageal and colon cancers [27]. The most recent study by our group, investigated the effects of DCA and CDCA in hypoxia induced prostate and breast cancer models, where HIF-1α was constitutively activated. We observed significant reductions in cell invasion, cell migration, cell adhesion and cell survival (clonogenicity) in the presence of physiological levels of bile acids, without inducing cytotoxicity or apoptosis [28]. Concomitantly, we observed that HIF-1α was significantly destabilised (when compared to controls) and the expression of its downstream signalling effector hexokinase II (HKII) was also reduced.

**Table 2:** The potentially therapeutic impact of bile acids on cancer development

Bile Acid	Signal Pathway/Molecular basis	Bile acid modulated cancer	Reference
**Lithocholic acid (LCA)**	Promotes cleavage of Bid and Bax and down-regulation of Bcl-2. No effect on prostate cell proliferation	Prostate Cancer	Goldberg *et al* (2013),
**CDCA and DCA**	HIF-1α destabilisation and significant reduction in cancer invasion phenotypes	Prostate and Breast Cancer	Phelan *et al* (2016)
**CDCA and DCA**	HIF-1α destabilisation	Metastatic Cancer	Legendre *et al* (2014)
**DCA**	DCA induction of MUC2 and inhibition of cell invasion and migration, reduction in Snail and MMP9 expression	Gastric Carcinoma	Pyo *et al* (2015)
**Bile acids**	Inhibition of HL60 and THP-1 proliferation, retinoic acid and vitamin D in differentiation, and down-regulating the serine protease myeloblastin	Leukaemia	Zimber *et al* (1994) Zimber *et al* (2000)
**UCDA**	Derivatives arrested cell cycle at G1, suppressed cdk2 and E-dependent kinase activities,	Prostate Carcinoma	Xu et al (2017) Choi et al (2003)

For some cancers, bile acids appear to suppress cancer invasion phenotypes and may destabilise HIF-1α, a key regulator of hypoxia in metastatic disease.

These studies exemplify an increasing awareness of the suppressive effects (molecular and phenotypic) of bile acids in the evolution of some cancers. However, defining the roles of these molecules in cancer is not helped by complicated bile acid receptor expression profiles and signalling mechanisms at the nucleus and plasma membrane. In their comprehensive review, Baptissart *et al.,* proposed that bile acids may have paradoxical functions depending on tissue distribution and the relevant bile acid receptor [29]. For example, farnesoid X receptor (FXR) expression at the oesophagus may promote inflammation and apoptosis resistance, while opposite effects may be exerted in the liver and intestine. Equally, it has been shown that different bile acids (conjugated and unconjugated) can exert differential FXR expression in a cholangiocarcinoma cell model [30]. Notwithstanding this complex receptor biology, it has been proposed that high levels of FXR expression could be strong positive prognostic indicators in invasive breast cancer [31]. Similarly, high TGR5 expression may be associated with oesophageal adenocarcinoma but may have more complex functions in the liver and intestine [29]. Strategies to target specific receptors in specific organ types must be informed by the knowledge that these receptors are required for normal physiology e.g. FXR and its role in bile acid/cholesterol homeostasis in the liver [32]. However, potential therapeutic advances have been made in this area, e.g. the identification of a detoxifying role for FXR in colorectal cancer (CRC), the third most common malignancy worldwide [11].

### Factors shaping bile acid profiles

#### The bile acid/microbiome axis

While the molecular and cell behavioural events induced by bile acids in cancer development are important, it is vital to assess factors that shape and sculpt bile acid profiles. The role of intestinal microbiota-derived bile acid metabolites in the aetiology of colorectal cancer was hypothesised several decade ago [33, 34]. Understanding and dissecting the microbiome/bile acid axis will help identify how potentially pathophysiological bile acid profiles are generated in the body and more importantly, how they impact on cancer development.

The gut microbiome is a community of commensal, symbiotic and pathogenic microorganisms that inhabit the gastrointestinal tract contributing both to human health and disease. Dietary and environmental factors, lifestyle choices or other conditions that impact on the composition and metabolic activity of intestinal microbiota, can lead to dramatic alterations in bile acid pools which in turn affects host metabolism and homeostasis [35–38]. The gut microbiota regulate bile acid metabolism, including the synthesis, conjugation, uptake and recirculation to the liver via the portal vein through FXR modulation, as well as by the transformation of bile salts into secondary bile acids [39]. Activation of bile-responsive receptors are not only key for bile acid metabolism, but they also influence a host of metabolic processes including, glucose homeostasis, lipid and lipoprotein metabolism, energy expenditure, intestinal bacterial growth, inflammation, liver functions, and hepato-carcinogenesis [40]. On the other hand, bile acids can also configure the structure of intestinal bacterial communities by selecting bile tolerant microbes or by fitness gain conferred by specific bile acid profiles [41]. The picture becomes even more complicated when we consider additional components of the gut ecosystem such as the enteric bacteriophage community, which has been poorly characterised and may participate in gut response to bile acids. The enteric virome regulates gut microbial population dynamics contributing to the normal functioning and resilience of the gut community [42]. Differential bile acid tolerances have been reported for some bacteriophages [43, 44], suggesting that bile acid-mediated modulation of enteric bacteria could be secondary to changes in bacteriophage diversity. Accordingly, recent studies have shown that the distinctive compositional signatures of gut bacteria linked to different human conditions are associated with specific enteric viromes [42]. Thus, different host-derived bile acid profiles may also alter bacterial composition and the production of secondary bile acids, by selecting for singular phage populations.

The gut microbiota also regulates inflammatory responses in the host by controlling the production of specific secondary bile acids such as LCA (lithocholic acid) or DCA, which activate the bile-responsive receptors FXR and TGR5. Activation of FXR or TGR5 results in an anti-inflammatory response by supressing the NF-kB-mediated production of pro-inflammatory cytokines in immune cells and the Caco-2 intestinal epithelial cell line [45, 46]. Accordingly, the dysregulation of bile acids has been implicated in several human diseases associated with inflammation, e.g. liver cirrhosis [7, 52], inflammatory bowel disease [45, 46, 47] type −2 diabetes (T2DM) [53] and cardiovascular disease (CVD) [54, 55]. These conditions have been associated with specific gut microbiota compositional and/or functional profiles [45, 56, 57]. This suggests that events at the microbiome have far reaching consequences and are not just limited to host metabolic disorders [50, 51]. For an in-depth and comprehensive review on the role of bile acids in metabolic disorders, refer to Jones *et al.* [5].

**Figure 1:**
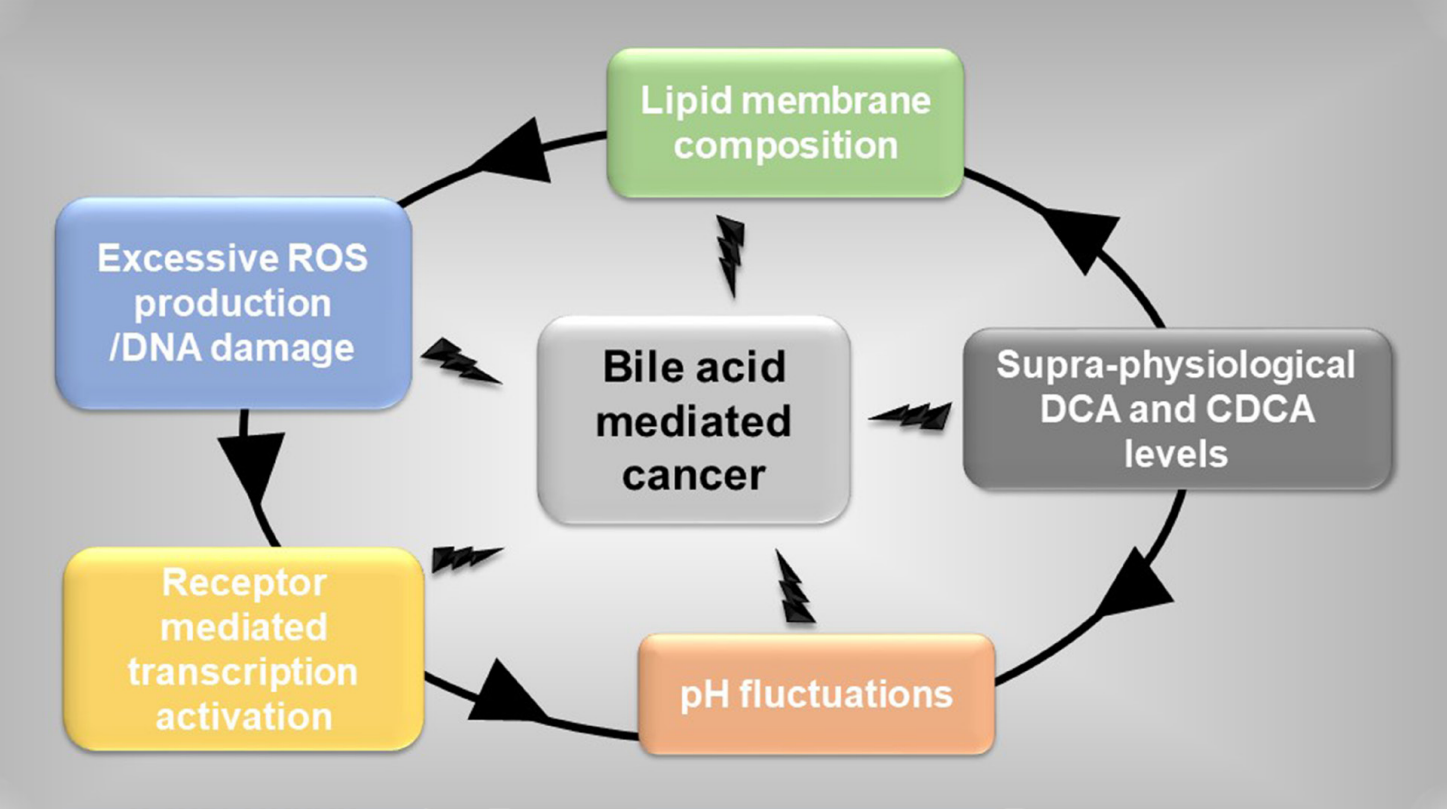
Events initiating bile acid mediated cancer Supra-physiological levels of bile acids exacerbate key cellular physiological events in cancer development.

**Figure 2:**
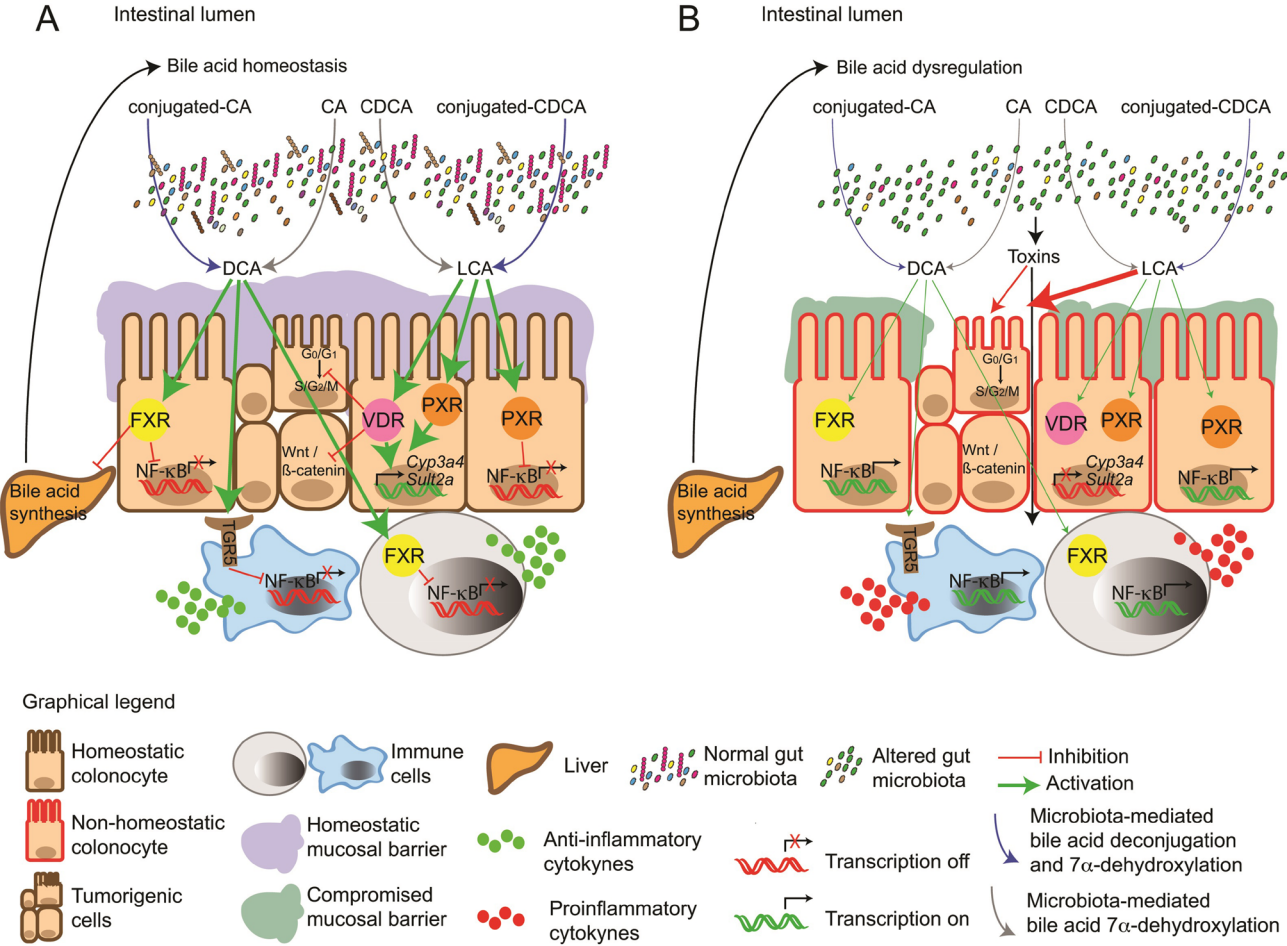
Molecular events arising in response to (**A**) bile acid homeostasis and (**B**) bile acid dysregulation. (A) Unaltered gut microbiota enzymatically modify primary bile acid pools (conjugated and unconjugated bile acids) to unconjugated secondary bile acids DCA and LCA. Secondary bile acids activate the nuclear receptors FXR, PXR and the G protein-coupled receptor TGR5 preventing NF-kB-mediated production of pro-inflammatory cytokines. FXR activation also inhibits bile acid synthesis in the liver and maintenance of bile acid homeostasis. LCA can also induce the (Vitamin D-Receptor) VDR and PXR-mediated transcription of genes involved in the detoxification and clearance of toxic metabolites such as LCA. (B) Factors affecting gut microbiota can alter the bile acid/gut microbiome axis, generating pathophysiological levels of bile acids and bile profiles that no longer activate FXR, PXR, VDR and TGR5, facilitating NF-KB-mediated expression of pro-inflammatory cytokines. Production of toxins or harmful metabolic by-products by dysbiotic gut microbiota also stimulate production of inflammatory markers by immune cells, which in turn contributes to disruption of the epithelial barrier. Downregulation of genes involved in the clearance of harmful metabolites, contributes to the accumulation of hydrophobic secondary bile acids in the intestinal lumen, which can induce damage in the cell membrane. The absence of FXR-mediated signalling results in the loss of inhibition of bile acid synthesis in the liver, contributing to a vicious cycle that increases levels of bile acids in the intestinal lumen. This exacerbates a pro-inflammatory phenotype. Together with excessive ROS production and localised genomic instability, preliminary neoplasms develop in vulnerable tissues and organs e.g. oesophagus, intestine and colon.

What precisely causes these pathological manifestations remains to be fully delineated. For cancer, bile acid mediated initiation appears to be promoted through several factors (individual or combined) (Figure 1); i) supra-physiological levels of bile acids (DCA) linked to poor nutrition, ii) lipid membrane composition at susceptible organs [25, 58], iii) ROS-mediated damage at epithelium barriers between host and microbiome, generating inflammation and hyper-proliferative epithelium phenotypes [6], iv) excessive pH fluctuations (e.g. due to over reliance on medicines) causing resurgences of pathogenic microbial populations [8] and v) activation of transcriptional programmes regulated by different nuclear receptors and altered production of cytokines that induce pro-inflammatory responses [11, 45, 59]. Unlocking the molecular interplay between these factors and their association with elevated bile acids (excessive DCA production) and cancer progression could shed light on the altered expression of key biochemical messengers, e.g. NF-kB and FXR (Figure 2), that when combined not only promote localised epithelium damage but also early stages of colorectal cancer [2].

Diet appears to be a pivotal factor in controlling bile acids. DCA levels are controlled by the bile salt hydrolysing *Roseburia* and *Faecalibacterium* to maintain BA pool sizes and composition. Fibre rich diets stimulate the growth of both *Roseburia* and *Faecalibacterium* species as well as promoting bile acid metabolism [60, 61]. Deficiencies in these genera can therefore upset DCA production and increase inflammation, leading to ulcerative colitis, a precursor to colon cancer [45, 62]. DCA production can become elevated thanks to high fat diets, inhibiting the growth of particular taxa of the phyla *Bacteroidetes* and *Firmicutes*, causing dysbiosis, e.g. inflammatory bowel disease, a potential precursor to colorectal cancer [63, 64].

It is a sobering thought that only a small fraction of all microbes are easily cultured and characterised in the laboratory [65]. Concerted efforts should be made to study and characterise microbiome strains that have been implicated in cancer. Strains such as *Enterobacteriaceae* that are associated with bile acid fluctuations in liver cirrhosis, and bile modulating strains such as *Clostridia* and its reported association with liver cancer development, require complete genotyping and characterising to delineate their precise roles in vulnerable cancer-susceptible individuals and cohorts [7, 8]. Significant efforts have yet to be made in this area, however Ahern *et al* provide a valuable procedural template in their quest to mine microbiota strains implicated in the immune system [66]. They suggest approaches including, culturing using complex media, dysbiotic gut transplantation into germ free mice, generation of strain libraries and intensive low error amplicon 16sRNA sequencing (LEA-Seq).

### The diet

The adoption of busier lifestyles means that “healthy” diets are often side-lined, leading to compromised eating habits with detrimental effects on health [67]. Eschewing healthier options sustains diets high in processed sugars, complex carbohydrates and saturated fats which complicate disorders such as diabetes, obesity and CVD. When seditious lifestyles are included, health and socio-economic issues become critical, leading to an overweight, chronically unfit population prone to deleterious ill health and potentially long term debilitating disease [68–71].

Several reviews suggest the consumption of red meat and dietary fats may be direct contributory factors to the development of cancers at e.g. the colon and liver [9, 72, 73]. Similarly, dairy products containing bile acid hydrolysing (BSH) genera (*Lactobacillus* and *Bifidobacterium)* should be carefully evaluated in terms of their potential impact on vulnerable cancer patients or sur viv ors [74]. BSHs have traditionally been identified as effective probiotics in the modulation of metabolic disease [5, 62], however BSH metabolism could lead to the generation of unwanted unconjugated secondary bile acids, exacerbating DNA instability at the colon [75]. Restoring normal bile acid profiles could be mediated by supplementing diets with probiotics (strains from the *Lactobacillus* and *Bifidobacterium* genera), prebiotics or non-digestible foodstuffs (inulin and *trans*-galacto-oligosaccharides) that would improve bile metabolic regulation and help readdress beneficial gut fauna [8, 76]. Much speculation exists in the literature regarding the use of probiotics and prebiotics against e.g. pancreatic, colon and colorectal cancer [77–80]. There is some evidence to suggest that secreted metabolites derived from *Lactobacillus* strains reduce the viability of colon cancer cells (Caco-2 and HT-29). These metabolites have been shown to downregulate the key receptor tyrosine kinases, Erb-2 and Erb-3, however the physiological implications of these findings have yet to be determined [81]. It is evident from the dearth of information in the literature that more research is required to fully realise the potential of microbial based therapies in restoring bile acid homeostasis in cancer cohorts where recurrence of disease is common.

While the generation of novel probiotics and microbiota-based strategies could potentiate bile signalling in cancer progression, there remains the possibility that control of bile acids from a dietary perspective could decrease susceptibility to cancer. Non-empirical evidence suggests that Mediterranean diets (fruits and vegetables, whole grains, legumes and nuts, olive oil, herbs and spices, limited red meat, fish and poultry and red wine (in moderation) could provide “probable” long term protective roles against cancer initiation [82]. However, while empirical data from a human perspective is not available [35, 48], it would be informative to conduct well designed and controlled, randomised, longitudinal bile acid (supplemented via probiotics or diet) studies in cohorts that are susceptible to colon, oesophageal or gastric cancers.

### Bile/Microbiome mediated anti-cancer therapies

Targeting and reducing excess bile acids without affecting normal signalling should be the main goal of research in this area. The advent of high throughput next generation sequencing methods targeted at bile acid producing bacteria could dramatically improve clinical diagnostics in cohorts where microbiome-related issues prevail [83]. For example, limited spectrum and non-absorbable antibiotics (e.g. ampicillin, vancomycin, neomycin and metronidazole) could target DCA mediated genotoxicity, generated by DCA producing bacteria (e.g. *Costridia*) in high risk colon cancer patients [8, 73]. Similarly, DCA levels are reduced in mice by decreasing 7α-dihydroxylation activity using fructose anhydride III or increasing bile acid secretion through ursodeoxycholic acid (UCDA) administration [84]. UDCA has long been speculated to act as a potential therapeutic agent in the treatment of colorectal and liver cancer. Chronic UDCA enrichment of rodent diets decreased DCA pools and decreased the incidence of benign and malignant tumours in these animals [2]. Similarly, UCDA and di-fructose anhydride III was shown to block DCA production in obese mice, preventing hepatocellular carcinoma in these animals [73]. UCDA appears to have potentially chemotherapeutic effects in humans. In a human phase III trial, UDCA administration was associated with significant reductions in the recurrence of colorectal adenomas with high-grade dysplasia in 1285 patients, a key finding for those prone to invasive colorectal carcinoma [85]. The generation of a synthetic derivative of CDCA (HS-1200) has been shown to be effective in inhibiting proliferation of prostate cancer cells, bone sarcoma cells and hepato-carcinogenesis in rats [86, 87]. Lastly, the use of the synthetic acid derivative obeticholic acid, a strong FXR agonist has been demonstrated to be a highly effective treatment for patients with primary biliary cirrhosis [88]. The efficacy of obeticholic acid in patients with primary biliary cirrhosis with inadequate responses to ursodeoxycholic acid could be a promising therapeutic option for treating liver disease.

Equally, microbiome transplantation, traditionally used for alleviating *Clostridium difficile* infections, has proven successful for some patients with colitis (precursor to cancer). The approach, however should be viewed with caution as pathogen and virus transmission to new hosts could induce host-specific microbiome immune effects [8, 76]. Notwithstanding these difficulties, microbiota transplantations appear to have universal appeal as therapies in the treatment of disorders such as microbiota-brain-gut axis dysregulation, metabolic and mood disorders. Therefore, their use as potential cancer therapies is a distinct possibility [49]. Similarly, future studies on non-bile modulating organisms could be used to regulate gut dynamics to protect against bile acid producing pathogens implicated in colon cancer. In support of this, preliminary research in predisposed mice, has shown that a *Lactobacillus acidophilus* strain deficient for lipoteichoic acid (LTA) regresses colonic polyps, a precursor to colon cancer [89]. Could genetically modified *L. acidophilus* strains provide protective effects against pathophysiological bile acid levels in susceptible colon cancer cohorts? For liver cancer susceptibility in obese cohorts, continuous monitoring of serum and faecal DCA levels could prove effective in predicting obesity associated liver cancer [90]. Supplementation of antioxidant agents to limit DCA-dependent ROS cell membrane damage has also been proposed as a potential therapy for Barret’s oesophagus patients [14, 91]. Similarly, aspirin could exert chemo-preventative effects on key apoptotic signalling molecules at different organ sites; e.g. in human gastric carcinoma cells, aspirin prevented DCA induced caspase-3, -6 and -9 activation, DNA degradation, poly (ADP-ribose) polymerase and lamin A processing [18]. In an oesophageal adenocarcinoma cell line, aspirin decreased MUC2 transcription and NF-κB activity [19]. Whether these pre-clinical observations can be translated to human studies remains to be seen.

## CONCLUSIONS AND PERSPECTIVES

The key genomic fault-lines that underpin cancer progression; intrinsic DNA/genomic instability, high cell turnover and regulation of cell survival/apoptosis/necrosis are chronically exacerbated by persistent supra-concentrations of bile acids at e.g. the colon, oesophagus and stomach. However, reports have shown that bile acids can have therapeutic effects on some cancer cell types, eliciting dramatic changes in cancer cell characteristics (cell adhesion, cell migration and cell invasion). How these effects are elicited remains to be elucidated, but it is tempting to speculate that bile signalling may have implications for hypoxic metastatic tumour development where HIF-1α is destabilised and expression of its effector, hexokinase II (HKII) is decreased [26, 28].

It has been observed that dysbiosis occurs in many inflammatory diseases, suggesting that defects in inflammatory signalling could contribute to these disease states [35]. Bile acids not only target and destabilise HIF-1α, but they induce the differential expression of the interleukins, IL-6 and IL-8 in airway epithelial cells [92]. These findings are important for two reasons. Firstly IL-8 mRNA expression is increased in colorectal cancer and secondly IL-8 mRNA expression is decreased in an airway epithelial cell model [92, 93]. It is thus tempting to postulate that excessive circulating bile acids induce differential inflammatory responses contributing to downstream epithelium hyper-proliferation in the colon and oesophagus.

**Figure 3:**
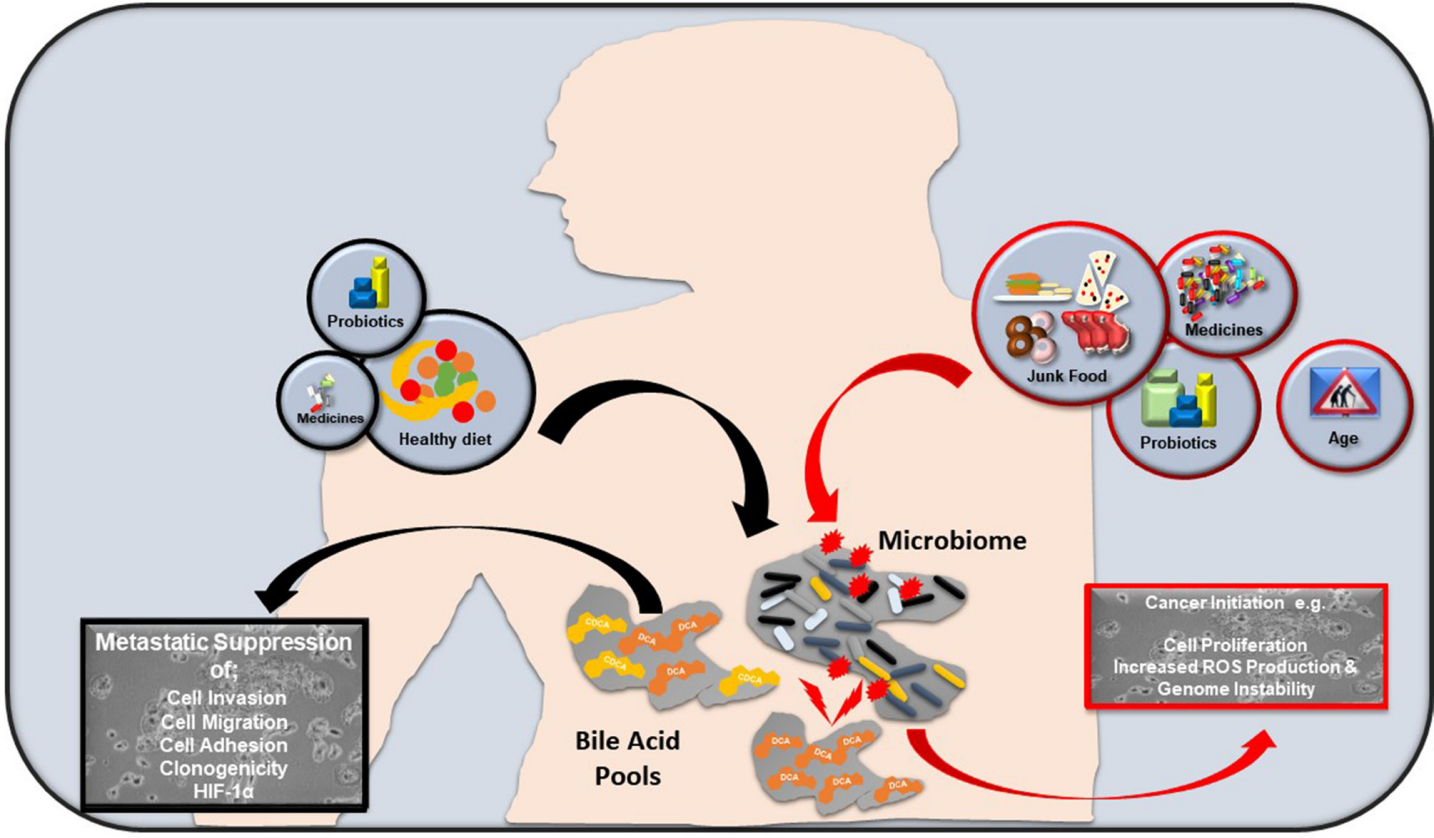
How bile acids may influence cancer progression Factors incorporating an unbalanced approach to diet (red line), junk food, excessive medicine/probiotic intake and age affect bile acid production. This alters the bile acid/gut microbiome axis, generating pathophysiological levels of bile acids. Together with excessive ROS and localised genomic instability, preliminary neoplasms can develop in vulnerable tissues and organs e.g. oesophagus, intestine and colon. Conversely, a healthy approach to diet (black line), appropriate medicine intake and other factors may help reduce pathophysiological bile levels, thereby maintaining a healthy bile acid/ gut microbiome axis. If potentially metastatic lesions are present in susceptible organs, physiological bile acid levels could limit cell invasion, cell migration and cell adhesion phenotypes.

The role of bile acid signalling in cancer progression is not a singular event; diet, probiotics, medicine, aging and the environment shape the bile acid/microbiome axis, therein determining bile acid levels and profiles. An unbalanced approach to diet and medicine intake may elevate bile acid levels and alter bile acid profiles to pathophysiological levels. This contributes to cell membrane damage and DNA instability through excessive ROS production. Downstream activation of important regulatory and inflammatory pathways (PKC, NF-κB, EGFR etc.) may lead to or exacerbate neoplastic cell proliferation in vulnerable organs e.g. oesophagus, stomach and colon (Figure 3). Conversely, a healthy balanced approach to diet, medicine intake and other factors may contribute to physiological levels of circulating bile acids, thus reducing pathophysiological bile levels. If a pre-cancerous lesion (with metastatic potential) is present in a susceptible organ, physiological bile acid levels could, based on the data presented here, limit progression of malignant cells (Figure 3).

In conclusion, the bulk of the literature promotes an associative relationship between bile acids and some cancers reinforcing a “foe” element of bile acids and its role in cancer. However, there remains the distinct possibility that some bile acids can elicit anti-cancer phenotypes (the “friend” element) in cells that have already undergone an initial malignant transformation (e.g. through HIF-1α activation). The maintenance of a balanced bile acid/microbiome axis could limit the metastatic potential of some cell types. Characterisation of this axis requires high-throughput metagenomics-sequencing approaches, intensive identification methods using the latest 16sRNA technologies, molecular based studies and practical, well-conceived human randomised trials. These approaches may provide some answers to the following questions; i) how do bile receptors in different tissues respond to bile acids, ii) what are the downstream targets of this regulation and can these bile receptors be targeted in those susceptible cancer cohorts with a dysfunctional bile acid/microbiome axis profile, iii) can harmful bile modulating gut pathogens be replaced by beneficial ones? While these questions remain to be answered, the solution for most lies in the diet. Promoting healthier eating habits could, in the long term, reduce predisposition to certain cancers where bile acids are perceived as contributory agents to disease.
